# Elcatonin attenuates disuse osteoporosis after fracture fixation of tubular bone in rats

**DOI:** 10.1186/s13018-015-0246-0

**Published:** 2015-07-03

**Authors:** Zhe Ji, Chao Shi, Shengli Huang, Xiaoqian Dang, Kunzheng Wang, Binshang Lan

**Affiliations:** Department of Orthopaedics, The Second Affiliated Hospital, School of Medicine, Xi’an Jiaotong University, Xi’an, Shaanxi Province 710004 People’s Republic of China

**Keywords:** Elcatonin, Disuse osteoporosis, Bone fracture, Fixation

## Abstract

**Background:**

Elcatonin (ECT) is used to prevent and treat osteoporosis. However, little is known about its effect on the disuse osteoporosis (DOP). The aim of this study is to evaluate the effect of ECT on DOP caused by fracture fixation.

**Methods:**

Forty-five male Sprague-Dawley (SD) rats, aged 6 weeks, were randomly allocated into three groups: the control group without surgery and elcatonin treatment (CTR, *n* = 15), the surgery group without elcatonin treatment (SUR, *n* = 15), and the surgery group which received elcatonin subcutaneously (SUR + ECT, *n* = 15). Surgery was produced by cutting the midshaft of the right femur transversely, fixing with stainless intramedullary needle, and immobilizing the right leg. All the proximal tibias from the random five rats in each group were harvested and investigated by evaluating bone mineral density (BMD), X-ray images, and histological staining respectively at the 4th, 8th, and 12th weeks after surgery.

**Results:**

Both of the SUR and SUR + ECT groups obviously exhibited lower BMD values compared to the CTR group; however, the SUR + ECT group showed significantly higher BMD values (*p* < 0.001, *p* < 0.05, and *p* < 0.05) than the SUR group at each time point after surgery. Moreover, similar changes were observed between these groups when examining the radiographs and hematoxylin and eosin (HE) staining.

**Conclusions:**

Elcatonin attenuates disuse osteoporosis after fractures in rats, which may provide a new avenue to prevent and treat disuse osteoporosis after surgery in clinic.

## Introduction

Disuse osteoporosis (DOP) is defined as localized or generalized bone loss due to skeletal mechanical unloading [1–3], such as long-term therapeutic bed rest [4], immobilization due to motor paralysis from injury of the central nervous system [5] or peripheral nerves, application of cast to treat fractures [6], and prolonged weightlessness during spaceflight [7]. In clinic practice, DOP is inevitable when plaster fixation is applied to stabilize the fracture and immobilize the bones or joints after the surgery [3, 6], which results in a decrease of bone strength and an increased risk of secondary fracture. Since the pathophysiology of DOP is still unclear, the treatments, for instance, therapeutic exercise, electrical therapy, calcium, vitamin D, and bisphosphonates, are not quite satisfactory so far. However, it is reported that low or loss of mechanical stress on bones induces acceleration of osteoclast-mediated bone resorption and inhibition of osteoblast-mediated bone formation and finally leads to bone loss [1, 3, 8]. Therefore, anti-resorptive agents were considered to prevent or treat DOP.

Elcatonin (ECT) is a physicochemically and biologically stable synthetic derivative of eel calcitonin and which consists of 31 amino acids with stable ethylene linkage as a result of substituting the intra-chain disulfide bond of calcitonin [9, 10]. It has been reported that ECT suppresses bone resorption through direct action on the calcitonin receptors of osteoclasts [11, 12] and acts with potent analgesic effect [12–15]. Moreover, data showed that ECT did not delay the overall fracture healing process compared to alendronate which strongly suppressed callus remodeling in cynomolgus monkeys [9]. ECT and salmon calcitonin have been worldwide used for the prophylaxis and treatment of osteoporosis [14, 16, 17], but a beneficial effect of ECT on bone has not been evaluated in surgically induced DOP yet.

Therefore, in the present study, we investigated the effects of the administration of elcatonin on the distal bone away from the fracture fixation of femur in rats which were rodent models of surgically-induced DOP.

## Materials and methods

### Animals

Fifty male Sprague-Dawley rats, aged 6 weeks in this study, were provided by Laboratory Animal Center of Xi’an Jiaotong University. All rats were housed individually in cages in a standard animal room maintained at a temperature of 22 ± 3 °C, a humidity of 50 ± 20 %, and a 12-h light-dark cycle, and ventilated ten or more times per hour. The animals were allowed access to food and tap water ad libitum during the entire experiment period. All animal procedures were conducted in accordance with the Principles of Laboratory Animal Care established by National Institutes of Health (NIH) and guidelines from Laboratory Animal Care Committee of Xi’an Jiaotong University.

Besides the five rats that were harvested and followed by histological staining procedure before surgery, other 45 animals were randomly allocated into three groups with 15 each: the control group without surgery and ECT treatment (CTR), the surgery group without ECT treatment (SUR), and the surgery group which received ECT subcutaneously (SUR + ECT). Body weight of the animals was recorded monthly. Surgery was conducted after 3-day feeding by cutting the midshaft of the right femur transversely, fixing with stainless intramedullary needle, and immobilizing the right hind limb via tibia-tail fixation. All the femurs, knee joints, and tibias from the random five rats in each group were respectively harvested at the 4th, 8th, and 12th weeks after surgery, and the proximal tibias were investigated by evaluating bone mineral density (BMD), X-ray images, and histological staining.

### Establishment of the animal model

The surgeries including post-osteotomy fixation and tibia-tail fixation were performed under anesthesia with 10 % chloral hydrate administered at a dose of 50 mg/kg, intraperitoneally.

1. Post-osteotomy fixation: The osteotomy and fixation were made in the right femur of each rat according to the method of Mathavan et al. [18]. Under standard sterile conditions, a lateral approach at the middle of femur was made and the periosteum was circumferentially incised. A transverse fracture was performed at the middiaphysis with an oscillating power saw, then the fracture site was stabilized and fixed by an intramedullary steel needle with a diameter of 1.2 mm (Fig. 1a). The incision was precisely closed with sterilized sutures.

**Fig. 1:**
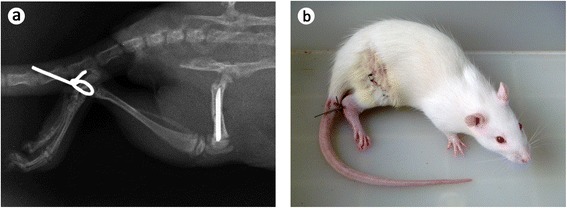
A radiographic capture and a camera photo of rat after surgical procedure. **a** The right femur was fractured and fixed with a steel needle, and the right hind limb was immobilized against the tail. **b** The rat can stand and move with other three legs after analepsia
2. Tibia-tail fixation: After the femoral fracture and internal fixation were accomplished, the right hind limbs of animals in the SUR and SUR + ECT groups were immobilized against the tails with simple external fixation based on the operation previously described by Zarzhevsky et al. [19]. Briefly, under anesthesia, the right ankles of animals were drilled, and a steel wire with diameter of 1.0 mm was inserted and connected to the tail at a distance of 1.5 cm from the anus. All rats were able to stand with their other three limbs after anesthesia resuscitation (Fig. 1b). The wires were removed 8 weeks after the surgery in the SUR and SUR + ECT groups. The external fixators were sterilized by iodophors and checked daily.

### ECT administration

Following the operations, ECT (Sidinuo, Luye Pharma Group Co. Ltd., Shandong Province, China) was administered at 15 U/kg three times a week [12] in SUR + ECT group, subcutaneously for 12 weeks. The CTR and SUR groups correspondingly administered the vehicles of the ECT injections.

### Evaluation methods

Bone samples from the right proximal tibias were subsequently evaluated via radiographic assessment, BMD measurement, and histology.

### Radiography

Antero-posterior radiographs were obtained from all animals via a clinical X-ray system at a condition of Voltage/Current = 52 kV/1.60 mA. The radiographs from the proximal tibias were visually assessed for the degrees of osteoporosis through double-blinded reviewing by two orthopedic surgeons. Compared to the CTR group, each bone from SUR or SUR + ECT group was scored as 0 (almost similar density), 0.5 (mildly lower density or thinner bone cortex), 1 (mildly lower density and thinner bone cortex), or 2 (severely lower density or/and thinner bone cortex).

### BMD measurement

BMD value was measured for the proximal third of right tibia which primarily consisted of cancellous bone via dual-energy X-ray absorptiometry (DXA) (Norland XR-46, Norland/Swissray, USA) at a scan pitch of 1.5 mm and a scan speed of 60 mm/s.

### Histology

Followed by tissue collection and decalcification with 10 % EDTA for 3 weeks, the right tibias from all groups were commenced with chemical fixation in 4 % formaldehyde for 48 h. Subsequently, the samples were dehydrated in graded ethanol, cleared in xylene, immerged in molten paraffin at 60 °C overnight, and embedded into wax block. Transverse sections of 4-μm thickness were cut by a microtome (RM2125RT, Leica Biosystems Nussloch GmbH, Germany) and stained with hematoxylin and eosin following standard protocol. The areas of bone trabeculae were quantified with Image-Pro Plus software (Version 6.0, Media Cybernetics, Inc., USA). Quantitative scoring of the histology was performed to determine the density of cancellous bone. Two blinded independent reviewers performed the analysis of all samples and the measurements were averaged.

### Statistical analysis

Data were expressed as the mean ± standard error of the mean (SEM). One-way ANOVA analysis was carried out to analyze for differences among the groups by SPSS (Version 22.0, SPSS/IBM Corporation, USA), and both Tukey HSD and Bonferroni tests were applied as a post hoc test if statistical significance was determined. A value of probability level (*p*) which was less than 0.05 was considered statistically significant.

## Results

### General condition

**Fig. 2:**
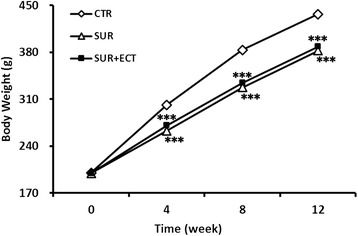
Body weight changes in the model of disuse osteoporosis. Data are expressed as mean ± SEM, *n* = 5. ***p < 0.001 versus the CTR group at each time point as evaluated by ANOVA

The animals survived well before harvesting, and no health problems were pointed out during the study period. As shown in Fig. 2, the body weight of rats in the CTR group was increased more than that in either of the SUR and SUR + ECT groups with obvious significant difference at different time point, and there was no statistical difference in the mean body weight between the SUR and SUR + ECT groups. However, the body weight gains in the SUR group was suppressed a bit than that in the SUR + ECT group at the 4th, 8th, and 12th weeks after surgery.

### Radiographic finding

**Fig. 3:**
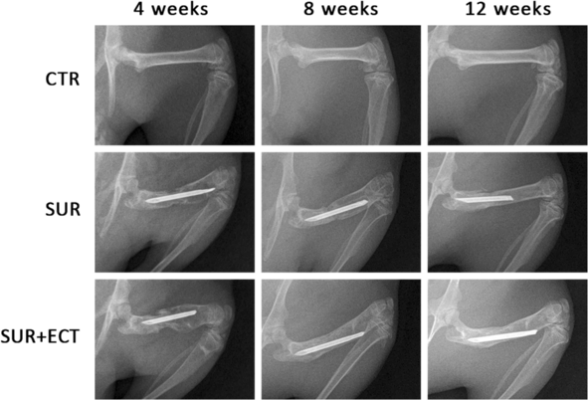
Representative radiographs of the femurs and tibias in the CTR, SUR, and SUR + ECT groups are presented at the 4th, 8th, and 12th weeks post-surgery, respectively

**Fig. 4:**
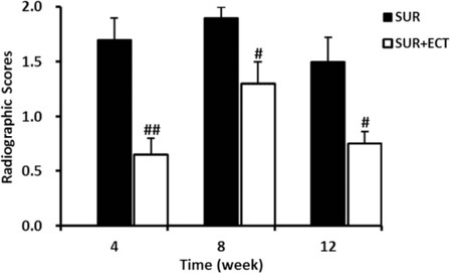
Radiographic scoring of osteoporosis in the proximal tibias by visual assessment by two orthopedic surgeons. Data are expressed as mean ± SEM, *n* = 5. #*p* < 0.05, ##*p* < 0.01 the SUR + ECT group versus the SUR group at each time point as evaluated by ANOVA

Compared to the CTR group at each time point (Fig. 3), both of SUR and SUR + ECT groups featured lower density and thinner bone cortex in the tibias, but much lower density and thinner bone cortex in the tibias in the SUR group existed than those in the SUR + ECT group. Moreover, compared to the SUR group, more bony callus formation, faster fracture healing, and higher density in the distal femur were observed in the SUR + ECT group at different time point. The scores of radiographs in the SUR group were significantly higher (*p* < 0.01, *p* < 0.05, and *p* < 0.05) at the 4th, 8th, and 12th weeks after surgery, respectively, compared to the SUR + ECT group (Fig. 4).

### BMD measurement

**Fig. 5:**
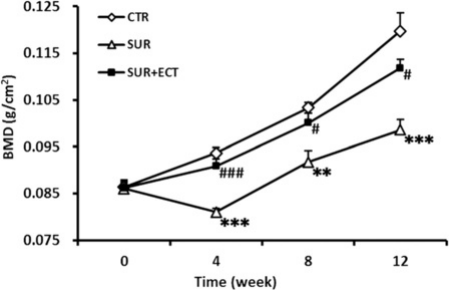
The BMDs of proximal third of right femurs in different groups at the 0th, 4th, 8th, and 12th weeks after surgery. Data are expressed as mean ± SEM, *n* = 5. **p < 0.01 and ***p < 0.001 versus the CTR group; #*p* < 0.05 and ###*p* < 0.001 the SUR + ECT group versus the SUR group at each time point as evaluated by ANOVA

In the SUR group, the BMD of proximal third of right tibia was obviously reduced (*p* < 0.001, *p* < 0.01, and *p* < 0.001) at each time point when compared with that in the CTR group (Fig. 5); and in particular, the BMD at the 4th week after surgery was significantly suppressed and decreased even below the BMD before surgery. Furthermore, in the SUR + ECT group, the BMD considerably increased (*p* < 0.001, *p* < 0.05, and *p* < 0.05) in the proximal third of right tibia when compared with that in the SUR group at each time spot, and the BMD of the proximal third was observed to be no significantly different from that in the CTR group.

### Histological scoring

**Fig. 6:**
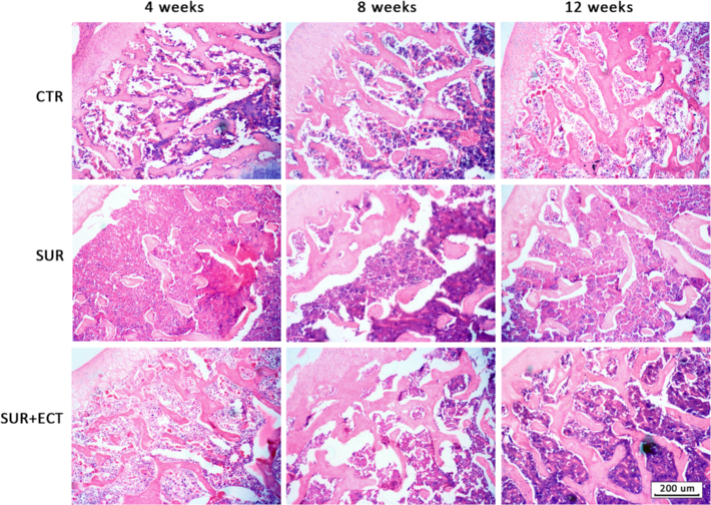
HE staining of proximal metaphysis of right tibia in the CTR, SUR, and SUR + ECT groups at the 4th, 8th, and 12th weeks post-surgery. Histological images were taken at 40× magnification

Analysis of the histological staining and scoring reinforced the findings of radiography and BMD measurement. As shown in Fig. 6, the bone trabeculae of the proximal metaphysis of right tibias in the SUR group were rare or vanished compared to the CTR group at the 4th week after surgery, and gradually getting more and better at the 8th and 12th weeks; however, the SUR + ECT group exhibited much denser and better arranged trabeculae than the SUR group at each time point and similar with the CTR group.

**Fig. 7:**
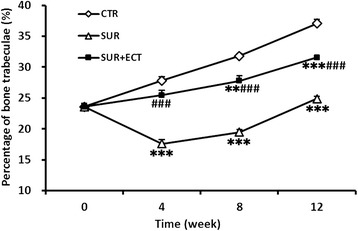
Quantitative histological scores of bone trabeculae from the proximal metaphysis of right tibia in the CTR, SUR, and SUR + ECT groups at the 0th, 4th, 8th, and 12th weeks post-surgery. Data are expressed as mean ± SEM, *n* = 5. **p < 0.01 and ***p < 0.001 versus the CTR group; ###*p* < 0.001 the SUR + ECT group versus the SUR group at each time point as evaluated by ANOVA

Moreover, according to the quantitative scoring of histology (Fig. 7), all animals that suffered surgery featured lower percentage of bone trabeculae during the whole research period compared to those in the CTR group except for the SUR + ECT group at the 4th week post-surgery. Additionally, the bone trabeculae were severely reduced in the SUR group at the 4th and 8th weeks (*p* < 0.001, respectively) even below the values of bone trabeculae at the 0th week, and the SUR + ECT group showed slower increases of bone trabeculae than the CTR group and significant differences (*p* < 0.001, *p* < 0.01, and *p* < 0.001) compared to the SUR group at each time point.

## Discussion

Using a well-established open rat model induced by post-osteotomy fixation and tibia-tail immobilization, our present study postulates and confirms the hypothesis that the administration of ECT attenuates disuse osteoporosis on the distal bone away from fracture fixation in rats. It is known that ECT is presently used worldwide for preventing and treating the osteoporosis, however, we were the first to investigate the effects of ECT on the post-surgery DOP, and three observations in particular demonstrated that ECT notably suppressed the bone loss at the 4th, 8th, and 12th weeks after surgery. The first observation was in relation to radiographic assessment where the SUR + ECT group exhibited higher density and thicker bone cortex in the proximal tibia compared to the SUR group. Radiographic outcomes is advantageous only if it is matched with BMD values, and this was substantiated with the second observation from the BMD measures where the values in the SUR + ECT group increased smoothly along with the CTR group. And quantitative histology showed an increased trabecular percentage in the SUR + ECT group compared to that in the SUR group. Thus, through manipulation of the anti-resorptive influences of ECT on surgery-induced DOP, an effective anti-osteoporosis outcome was achieved.

The technique of fracture fixation employed in our study [18, 20] is analogous to a common clinical method used in the treatment of femoral fractures, and the tibia-tail fixation [19, 21] is applied for establishing a model of unloading on the musculoskeletal system as the cast immobilization after fracture surgery in clinic.

In addition, the concerns of anti-osteoporosis effects by ECT have been interpreted in the literatures [9, 10, 22–24]. For instance, Iwamoto et al. presented data from a 5-year-period study demonstrating a sustainable role of ECT in post-menopausal women with osteoporosis [22]. In addition, another study in post-menopausal osteoporotic women confirmed the active effects of short-term combined treatment with bisphosphonate and ECT on lumbar BMD and bone turnover with back pain [23, 24]. Furthermore, mild suppression of callus remodeling by ECT did not impair overall fracture healing process in the femoral fracture model of cynomolgus monkeys [9], whereas ECT facilitated osteoblast proliferation in the fracture site and suppressed the systemic acceleration of bone resorption without affecting cortical bone regeneration after drill-hole injuries in mice [10]. According to the work above, it was considered to study the influence of ECT as an alternative chemical on the post-fracture DOP in rats without delaying the fracture healing.

During the 12 weeks of treatment, a marked lower body weight was observed both in the SUR and SUR + ECT groups (Fig. 2), which interpreted that the administration of ECT did not affect the weight changes of animals after surgery as body weight is a general index of the health of an animal [2]. And this might be attributed to a decrease in food intake after surgery.

As the plain X-ray film reveals important features of disuse osteoporosis affecting tubular bones, thinning of cortices, increased radiolucency, and rarefication of bone trabeculae [3, 18, 25], the potent anti-osteoporosis effectiveness of ECT in sustaining the bony density were clearly demonstrated in the results from the radiographic analysis where the presence of ECT produced less rarefication of bone in the proximal tibia at each time point. Moreover, with the addition of ECT to the animals after surgery, not only did the combination of fracture reliably and quickly achieve union but also did the features of bone substance in femurs attain nearly on par with the non-operated control femurs at the 12th week post-surgery, which was in line with the conclusion described previously [10].

Since the technology of dual-energy X-ray absorptiometry (DXA) has been used to measure regional BMD [3] which has been described as a surrogate measure of bone strength [26], BMD measurements have developed and provided the basis for diagnosing osteoporosis, predicting the risk of osteoporotic fracture, and evaluating the therapeutic effects of drugs [27]. In our study, the BMDs in the proximal third of tibia from the SUR group at the 4th, 8th, and 12th weeks were all much lower than those in the normal rat, at the same time, it was observed that the therapy with ECT could increase the BMDs to a statistically undifferentiated level as the CTR group. In particular, the BMD values in the proximal third of tibia from non-ECT-treated rats went down dramatically at the 4th week after the fracture operation and tibia-tail fixation. This indicates that the administration of ECT we used in present study can suppress the surgically-induced DOP effectively both in the former 8 weeks with immobilization and in the later 4 weeks without.

Similar to our observations above, histological staining and analysis of the metaphysis from ECT-treated tibias confirmed significant increases in the quantitative trabeculae at the 4th, 8th, and 12th weeks after surgery compared to the SUR group. Under the circumstance of immobilization, the osteoclast activities increased because of the decreased mechanical stimulus [2]. Although its precise physiological role in the rats is not well understood, so far it is known that ECT inhibits bone resorption by binding to the calcitonin-like receptors on the osteoclast membrane, thus, destroying osteoclast activities [12, 28]. Based on our findings, we speculate that ECT might suppress osteoclast effectively so that there is no excessive bone resorption in osteogenesis or bone remodeling after the surgery mentioned in our work. Therefore, ECT could not only decrease the bone resorption but also slightly promote the ossification in the first 4 weeks after surgery.

Additionally, based on radiological, BMD, and histomorphometric evaluation of proximal tibia away from fractured femur without immobilization from 8 to 12 weeks in rats treated with ECT, we concluded that ECT might still sustain the osteogenic process and at least had no negative effect on the intrinsic properties of femurs and tibias.

There are several limitations in the current study. Firstly, it is an exclusively in vivo experiment for prophylactic and therapeutical effects of ECT on the surgically-induced DOP in animal models. In addition, a limited number of time points prohibited a more detailed observation of the stages of DOP and the ideal time for administration of ECT. Finally, anti-resorptive agents are generally administrated for elderly patients with osteoporosis clinically, and the animal models should provide clinically related information. Therefore, the elderly animals should be utilized to further elucidate a more complete understanding of the effect of ECT on the DOP after the fracture fixation in the future.

## Conclusion

The present study highlights that elcatonin can attenuate surgically induced bone loss and deterioration of trabeculae in the distal bone from fracture in rats, which may serve as an alternative medicine for the prophylaxis and treatment of disuse osteoporosis after surgery in clinic practice. In the future study, the molecular mechanisms of related protein and cytokine regulation behind the results and examinations need further investigation.
